# Concentration-Dependent Effects of N-3 Long-Chain Fatty Acids on Na,K-ATPase Activity in Human Endothelial Cells

**DOI:** 10.3390/molecules25010128

**Published:** 2019-12-28

**Authors:** Roberta Cazzola, Matteo Della Porta, Sara Castiglioni, Luciano Pinotti, Jeanette A.M. Maier, Benvenuto Cestaro

**Affiliations:** 1 Department of Biomedical and Clinical Sciences “L. Sacco”, Università di Milano, 20157 Milano, Italy; 2 Department of Health, Animal Science and Food Safety, VESPA, Università di Milano, 20133 Milano, Italy

**Keywords:** endothelium, sodium pump, eicosapentaenoic acid, docosahexaenoic acid, membrane fluidity, lipid peroxidation

## Abstract

N-3 eicosapentaenoic acid (EPA) and docosahexaenoic acid (DHA) seem to prevent endothelial dysfunction, a crucial step in atherogenesis, by modulating the levels of vasoactive molecules and by influencing Na,K-ATPase activity of vascular myocytes. The activity of endothelial Na,K-ATPase controls the ionic homeostasis of the neighboring cells, as well as cell function. However, controversy exists with respect to the vascular protective effect of EPA and DHA. We argue that this dispute might be due to the use of different concentrations of EPA and DHA in different studies. Therefore, this study was designed to define an optimal concentration of EPA and DHA to investigate endothelial function. For this purpose, human endothelial cells were exposed for 24 h to different concentrations of DHA or EPA (0–20 μM) to study membrane fluidity, peroxidation potential and Na,K-ATPase activity. EPA and DHA were linearly incorporated and this incorporation was mirrored by the linear increase of unsaturation index, membrane fluidity, and peroxidation potential. Na,K-ATPase activity peaked at 3.75 μM of EPA and DHA and then gradually decreased. It is noteworthy that DHA effects were always more pronounced than EPA. Concluding, low concentrations of EPA and DHA minimize peroxidation sensitivity and optimize Na,K-ATPase activity.

## 1. Introduction

The Na,K-ATPase or Na^+^ pump (EC 3.6.37) is an ubiquitous membrane transport protein whose activity determines and maintains high K*^+^* and low Na*^+^* concentrations in the cytoplasm, generates a potential across the membrane and provides the driving force for secondary ion transport [1]. Na,K-ATPase consists of two subunits: A large ouabain-sensitive polypeptide (α) responsible for transporting ions and a smaller glycoprotein (β) needed for enzymatic activity. These subunits are closely associated with lipids that modulate the activity of the pump [2]. Ion homeostasis, that is maintained by Na,K-ATPase, is critical for numerous cellular functions and processes, including cell growth, differentiation, migration, contraction, secretion, and volume regulation [3]. At vascular level, Na,K-ATPase plays a key role in modulating blood pressure. Experimental evidence has shown that Na^+^ pump inhibition causes the contraction of smooth muscle cells [4,5,6] by promoting Ca^2+^ entry into the smooth muscle cells through the inversion of the Na^+^-Ca^2+^ exchange system. Endothelial cells modulate vascular tone by releasing vasoactive mediators [1] and by permitting active solute transport between lumen and sub-endothelium. In particular, an asymmetrical localization of Na,K-ATPase between luminal and abluminal membranes of endothelial cells [4,5] determines trans-endothelial ionic gradients. These gradients control ionic homeostasis and the performance of the neighboring smooth muscle cells [7], while transendothelial K^+^ and Na^+^ transport regulates the activity of myocytal Na,K-ATPase [8], crucial for the regulation of vascular tone.

Controversy exists about the role of n-3 eicosapentaenoic acid (EPA) and docosahexaenoic acid (DHA) in cardiovascular disease prevention and endothelial function [9,10,11]. Several human studies suggest that high levels of the n-3 long-chain (LC) polyunsaturated fatty acids (PUFAs) eicosapentaenoic acid and docosahexaenoic acid in membrane phospholipids reduce cardiovascular risk [12,13] and improve endothelial function [14]. N-3 LC-PUFAs seem to regulate blood pressure and improve vascular integrity by preventing vascular inflammatory and adhesion cascades, and by enhancing the generation and bioavailability of nitric oxide through upregulation and the activation of endothelial nitric oxide synthase [15]. Surprisingly, incubation with high concentrations of n-3 LC-PUFAs has been shown to decrease Na, K-ATPase activity of human umbilical vein endothelial cells [16] and sheep pulmonary artery rings [17]. However, the underlying mechanisms of the effects of EPA and DHA have not yet been fully clarified. Na,K-ATPase activity is regulated by several factors, including the availability of substrates [18], hormones [19], circulating endogenous inhibitors, such as cardiotonic steroids (ouabain, digoxin, etc.) [3], as well as the chemical-physical features of the surrounding membrane lipid micro-environment, and in particular, the degree of membrane fluidity, the ratio between phospholipids and cholesterol [20]. It is widely believed that lipids that increase membrane fluidity promote Na,K-ATPase activation, and, vice versa, those that stiffen the membranes reduce pump activity [20].

Membrane lipids, such as phospholipid species, their fatty acyl chain length and degree of unsaturation, and cholesterol content all contribute to the fluidity of the membranes. In general, low cholesterol content and high degree of unsaturation of phospholipid fatty acyl chains are associated with fluid membranes [21,22,23]. The most unsaturated PUFAs in mammalian cell membranes are the long chain polyunsaturated FAs (LC-PUFAs) of the n-3 and n-6 families, such as EPA (C20:5 n-3), DHA (C22:6 n-3) and arachidonic acid (ARA, C20:4 n-6) derived from the nutritionally essential linolenic acid (C18:3 n-3), and linoleic acid (C18:2 n-6), respectively. The LC-PUFAs of these two families compete for the enzymes needed for their synthesis from the C18 precursors as well as for the enzymes that synthesize their derivatives. A diet rich in n-3 PUFA, or a dietary supplementation of EPA and/or DHA, increases the concentration of these FAs in membranes at the expense of ARA and, consequently, increases the degree of unsaturation of membrane lipids. It also reduces the levels of vasoconstrictor lipid mediators originating from ARA [24,25]. Moreover, recent studies on lipid bilayers and cellular systems have shown that EPA and DHA influence the size and order of membrane lipid microdomain. However, the complex effects of n-3 LC-PUFAs on membrane remodeling still need to be clarified [26].

The higher number of double bonds predispose LC-PUFAs to both enzymatic and non-enzymatic oxygenation. Enzymatic oxygenation gives rise to a plethora of metabolites that modulate receptor signaling and gene expression [27,28,29], whereas the non-enzymatic oxidation or peroxidation determines LC-PUFAs degradation to cytotoxic products, including peroxides and aldehydes that can greatly alter the physicochemical properties of membrane lipid bilayers [28,30,31], also through a reduction of their fluidity [32]. Moreover, lipid peroxidation-derived aldehydes react selectively with proteins or enzymes containing SH groups such as Na,K-ATPase, altering their functions [33]. Since peroxidation potential of PUFA increases with the degree of unsaturation, the enrichment in n-3 LC-PUFAs could enhance membrane lipid susceptibility to peroxidation. Therefore, high concentrations of LC-PUFA on one hand could improve Na,K-ATPase’s activity through their fluidizing action on membranes, on the other hand, they could reduce pump activity by increasing peroxidation potential.

The controversy around the vascular protective effect of EPA and DHA might result from the different concentrations of these compounds used in different studies. Therefore, we designed the present study to investigate the effects of different concentrations of EPA or DHA on Na,K-ATPase activity and membrane chemical-physical parameters that could affect the activity of this ATPase, such as the degree of membrane fluidity and the susceptibility to lipid peroxidation in human microvascular endothelial cells (HMEC).

## 2. Results and Discussion

### 2.1. Incorporation of N-3 LC-PUFA in Confluent HMEC

Membrane phospholipid (PL) fatty acid composition results from both *de novo* synthesis (Kennedy’s pathway) and PL remodeling, through the deacylation-reacylation processes (Lands’ pathway). The extent of the contribution of these two pathways varies from cell-to-cell, and depends on how fatty acids are supplied to cells. Human endothelial cells incorporate both free fatty acids and fatty acid-albumin complexes added to culture medium [34]. Since free FA form micelles in solution and act as detergents in the presence of lipid membranes [35], we have supplemented HMEC with EPA or DHA complexed with human albumin. PUFA supplied to human endothelial cells as albumin complexes are readily incorporated into the cellular PLs and, after 24 h of incubation, were found mainly (95%) in membranes where they modify fatty acid composition of PLs primarily via the Lands’ pathway [34].

Since 18 h of incubation with lipids suffice to modulate Na,K-ATPase activity of human endothelial cells [16,36], we considered it reasonable to perform our experiments after 24 h exposure to EPA or DHA in HMEC.

**Figure 1:**
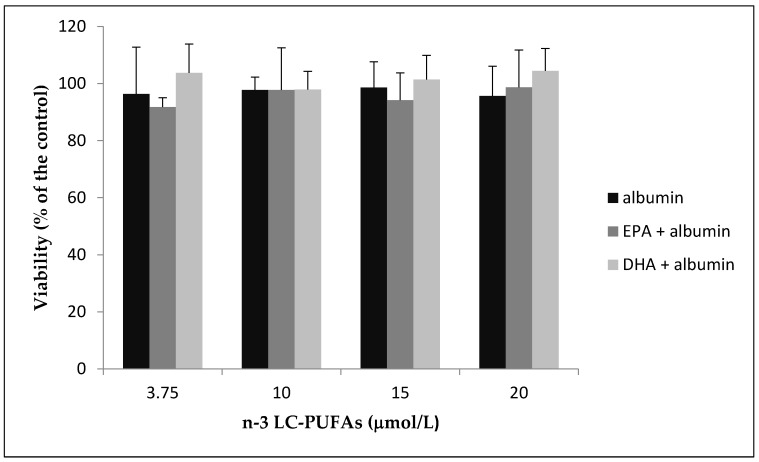
HMEC were incubated with increasing amounts of N-3 eicosapentaenoic acid (EPA) and docosahexaenoic acid (DHA) complexed with human defatted albumin or an equivalent amount of human defatted albumin (control) for 24 h. Viable cells were evaluated by MTT assay. Results from three separate experiments are shown as % of the control ± standard deviation.

As shown in Figure 1, 24 h of incubation with different concentrations of EPA or DHA did not affect cellular viability.

**Figure 2:**
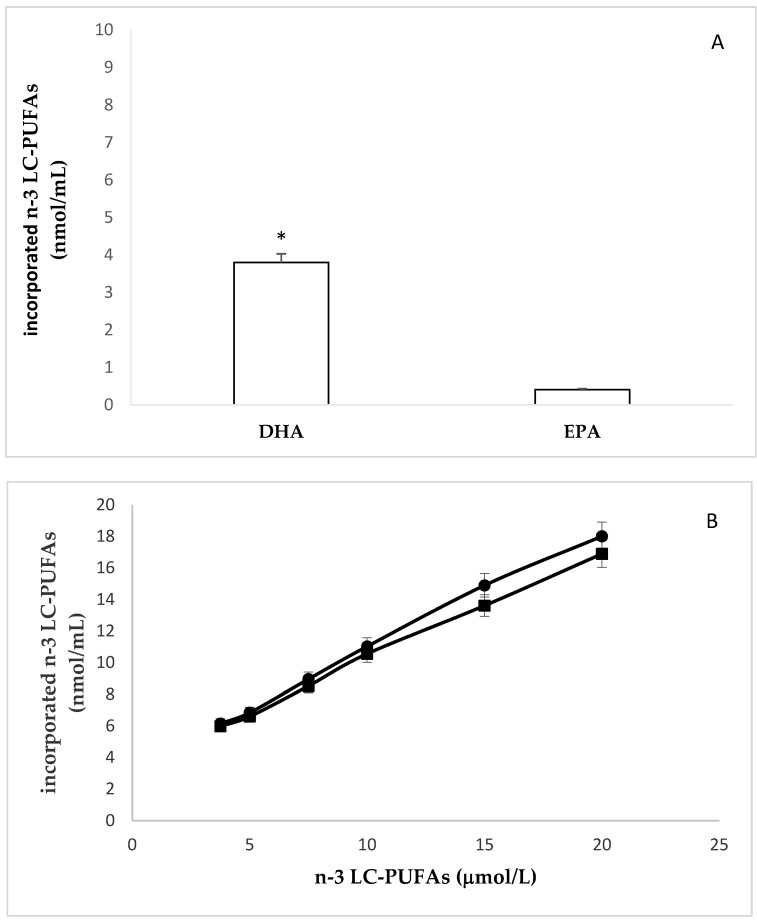

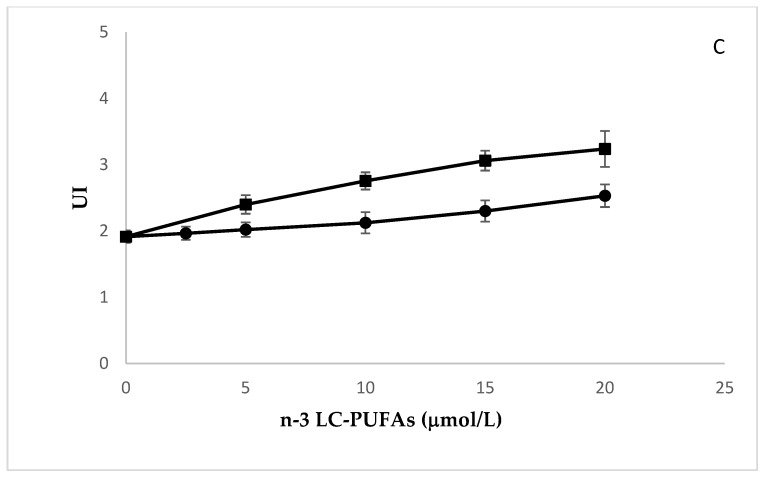
Incorporation of EPA or DHA in HMEC. (**A**): Basal levels of EPA and DHA. * EPA vs DHA *p* ≤ 0.001 (**B**): incorporation of EPA (●) and DHA (∎) after 24 h of cell incubation with increasing amounts of these n-3-LC-PUFAs. (**C**): Changes of unsaturation index (UI) after cell incubation with increasing amounts of EPA (●) and DHA (∎). Pearson’s r = 0.9999, *p* < 0.0001 and Pearson’s r = 0.9996, *p* < 0.0001. Slope EPA vs. slope DHA *p* ≤ 0.0001.

Cell lipids were then extracted, and the levels of EPA and DHA measured by gas chromatography. The incorporation of both fatty acids after 24 h of incubation is reported in Figure 2. We found significantly different amounts of EPA and DHA in unstimulated cells. Indeed, the basal levels of EPA and DHA were 0.408 ± 0.019 nmol/10^6^ cells and 3.8 ± 0.24 nmol/10^6^ cells, respectively (Figure 2A). The highest value incorporated of EPA and DHA were 18.0 ± 0.99 nmol/ 10^6^ cells, and 16.9 ± 0.97 nmol/ 10^6^ cells, respectively (Figure 2B).

In agreement with previous studies [34], fatty acid analysis of HMEC, enriched with EPA and DHA, revealed an increase of the ratio between saturated and polyunsaturated fatty acids, and a consequent increase in the concentration of double bonds in membrane phospholipids (unsaturation index, UI). As expected and depicted in Figure 2C, at each dose of n-3 LC-PUFA added to cell culture medium, DHA (six double bonds) determined a greater increase of UI than EPA (five double bonds).

All the experiments were repeated at least three times. Data are expressed as mean ± SD of replicates.

### 2.2. Effects of N-3 LC-PUFA Incorporation on Membrane Fluidity in HMEC

**Figure 3:**
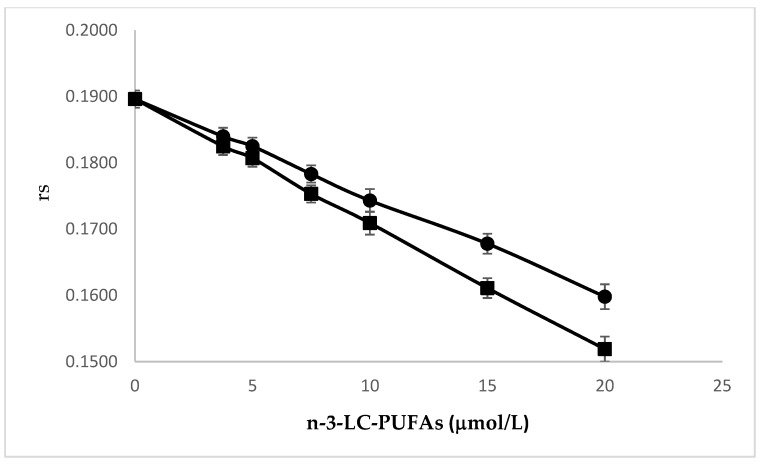
Effect of the incubation with increasing amounts of EPA (●) and DHA (∎) on fluorescence anisotropy (rs) of DPH in HMEC membranes. Experiments were performed in triplicate. Data are expressed as mean ± SD of replicates. Pearson’s r = 0.9999, *p* < 0.0001 and Pearson’s r = 0.9996, *p* < 0.0001; EPA and DHA, respectively. Slope EPA vs. slope DHA *p* ≤ 0.0001.

Membrane fluidity after incubation with EPA and DHA was evaluated in HMEC by measuring fluorescence anisotropy (rs) of 1,6-diphenyl-1,3,5-hexatriene (DPH), a hydrophobic compound almost non-fluorescent in water. When supplied to the cells, it is readily absorbed into the membranes where, after intercalation with PL acyl chains, it becomes fluorescent. Rs is a measure of the rotational mobility of this fluorophore when it is excited with polarized light. The higher the rs, the lower the fluidity, and vice versa. We found that the incorporation of increasing amounts of EPA and DHA fluidized membranes, as indicated by the corresponding rs decrease (Figure 3). Interestingly, DHA increased membrane fluidity significantly more than EPA. The study of correlations showed that the concentration of these n-3 LC-PUFA significantly correlates with rs (Pearson’s r = 0.9999, *p* < 0.0001 and Pearson’s r = 0.9996, *p* < 0.0001; EPA and DHA, respectively). The comparison of linear regression curves by one-way ANOVA showed significant differences between the slopes (*p* ≤ 0.0001). These results are in agreement with previous studies, showing that the unsaturation index (UI) of membrane phospholipids is one of the major factors influencing membrane fluidity [21,22,23]

### 2.3. Effects of N-3 LC-PUFA Incorporation on Peroxidation Potential in HMEC

**Figure 4:**
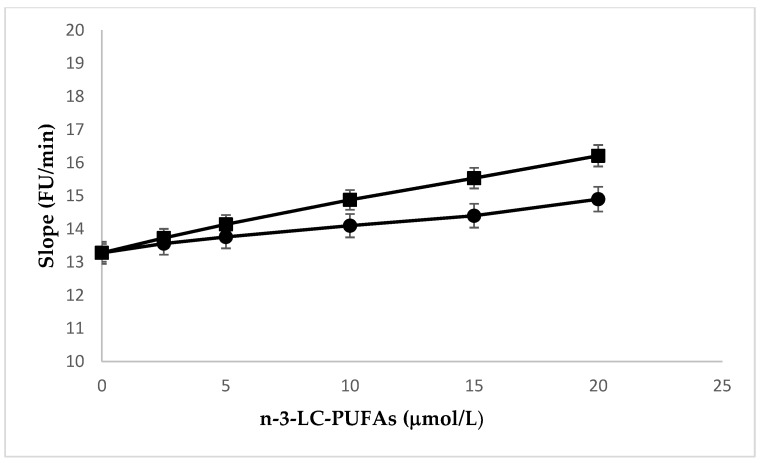
Effect of the incubation with increasing amounts of EPA (●) and DHA (∎) enrichment on peroxidation kinetics of HMEC. Experiments were performed in triplicate. Data are expressed as mean ± SD of replicates. Pearson’s r = 0.9921, *p* < 0.0001 and Pearson’s r = 0.9973, *p* < 0.0001; EPA and DHA, respectively. Slope EPA vs. slope DHA *p* ≤ 0.0001.

In confluent HMEC enriched with EPA or DHA, peroxidation was induced by a flux of aqueous peroxyl radicals derived from the thermal decomposition of 2,2′-azobis(2-amidinopropane) dihydrochloride (AAPH) and monitored by measuring intracellular oxidation of dichlorofluorescin diacetate (DCFD). The increase of EPA and DHA contents in the cells was associated with an increase in susceptibility to peroxidation, as shown by the concentration-dependent increase of the propagation rate (slope) of the kinetic peroxidation curves (Figure 4). The study of correlations showed that the concentration of these n-3 LC-PUFA significantly correlated with rs (Pearson’s r = 0.9921, *p* ≤ 0.0001 and Pearson’s r = 0.9973, *p* ≤ 0.0001; EPA, and DHA, respectively). The comparison of linear regression curves by ANOVA showed significant differences between slopes (*p* ≤ 0.0001). The concentration-dependent curves indicate that the effect of DHA was significantly higher than that of EPA.

AAPH is an extensively reported generator of free radicals that are physiologically relevant to biological systems [37,38]. The kinetic profile of peroxidation induced by AAPH is typically sigmoidal and characterized by a latency phase, a propagation phase, and termination [38]. In our experimental model, AAPH was added to culture medium, which contains antioxidants that protect the cells against peroxidation. Once the antioxidants are exhausted, AAPH radicals can attack the molecules of cell membranes. Given that PLs are more sensitive to these radical species than cholesterol and proteins [39], it is reasonable to propose that the PUFA of PLs are the first substrates to be oxidized, thereby promoting the initial formation of lipid peroxide in cell membranes. Lipid peroxidation of the membranes promotes the alteration of the redox homeostasis of cells and consequently the oxidation of DCFD. The compositional variation, due to the increased incorporation of n-3 LC-PUFAs, resulted in a higher concentration of carbon-carbon double bonds, which are the substrates of lipid peroxidation reactions. Moreover, the consequent increase in membrane fluidity renders PUFA easily accessible by the oxidants. As mentioned above, the incorporation of DHA increased IU and the fluidity of the membrane more than EPA.

### 2.4. Effects of N-3 LC-PUFA Incorporation on Na,K-ATPase

**Figure 5:**
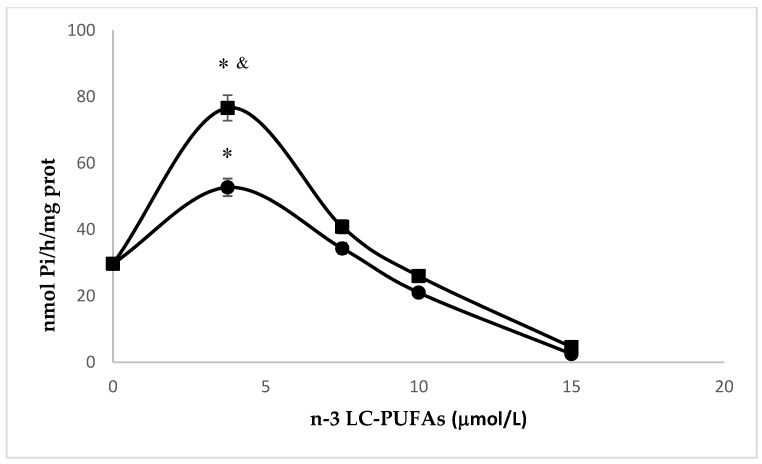
Effect of the incubation with increasing amounts of EPA (●) or DHA (∎) on Na,K-ATPase activity of HMEC. Experiments were performed in triplicate. Data are expressed as mean ± SD of replicates. The incubation of HMEC for 24 h with 3.75 μM of n-3 LC-PUFAs significantly increased pump activity when compared to basal levels and the other concentrations (*, *p* < 0.001, Student’s *t* test). DHA vs. EPA, *p* < 0.001, Student’s *t* test (^&^).

Na,K-ATPase in the endothelium was the topic of several investigations. In cerebral endothelial cells, the inhibition of the pump increased Ca^2+^ release from the endoplasmic reticulum, thus leading to endothelial injury [40]. Accordingly, in the present study, we tested the effect of EPA or DHA exposure on Na,K-ATPase activity in HMEC. As reported in Figure 5, the activity of the pump peaked with the lowest concentration of EPA and DHA utilized (3.75 μM) and then gradually decreased as the concentration of these n-3 LC-PUFA increased. Interestingly, DHA-induced peak was significantly higher than EPA’s (*p* < 0.001, Student’s *t* test). It is complex to translate these results to human physiology, because serum levels of these nutritional essential fatty acids are influenced by several factors, including genetic factors, diet, and lipemia. A few studies report the serum levels of EPA, ranging from approximately 1 μmol/L [41], to 400 μmol/L [42] while the concentrations of DHA varied between 2.3 μmol/L [41] and 580 μmol/L [42]. In our experimental model, the lowest concentration of n-3 LC-PUFAs added to culture medium was 3.75 μM, which falls within the physiological range [41,42], and determined a 3.5-fold and 1.5-fold increase of EPA and DHA, respectively, if compared to the basal level (*p* < 0.001, Student’s *t* test). Based on the concentration-dependent effects of n-3 LC-PUFAs, on all the parameters that we have measured, this seems to be the correct concentration to optimize the activity of Na, K-ATPase, since it exerts a fluidifying action without appreciably influencing peroxidability.

On the contrary, higher amounts of these PUFAs alter the chemical-physical properties of the membranes and inhibit the pump activity. Mayol et al. found that EPA and DHA inhibited Na,K-ATPase activity of macrovascular human endothelial cells by adding these n-3 LC-PUFAs to a culture medium at much higher concentrations (0.1 mM in form of emulsion with lecithin) than the ones we used in this study [16]. Similarly, Singh et al. found that 30 μM EPA inhibited pump activity in pulmonary vessel rings [17].

To the best of our knowledge, this paper is the first to describe a concentration dependent effect of n-3 LC-PUFA on Na^+^ pump activity in endothelial cells and to individuate a concentration that minimizes detrimental effects and optimizes pump activity.

## 3. Materials and Methods

### 3.1. Cell Culture

HMEC (LGC Standards-ATCC, Sesto S.G (MI), Italy) were grown in MCDB131 containing epidermal growth factor (EGF) (10 ng/mL), glutamine (2 mM) and 10% fetal bovine serum (FBS) on 2% gelatin-coated flasks. All culture reagents were from Gibco (Thermo Fisher Scientific, Waltham, MA, USA). HMEC grown to confluence were incubated for 24 h in the presence of increasing amounts (from 3.75 to 20 μM) of DHA or EPA (Sigma-Aldrich, Milan, Italy) in complex with human defatted albumin (Sigma-Aldrich, Milan, Italy) or human defatted albumin (control). To test cell viability, the MTT assay was utilized as previously described [43]. In other experiments, the cells were harvested for lipid extraction and subsequent determination of membrane fluidity, peroxidation potential and Na,K-ATPase activity.

### 3.2. Fatty Acids Analysis

To determine cell fatty acid composition, lipids were extracted in 2:1 chloroform/methanol (Sigma-Aldrich, Milan, Italy) containing 0.2% butylated hydroxytoluene (Merck, Darmstadt, Germany), according to the method by Folch et al. [44]. The lipid extracts were evaporated under nitrogen stream and trans-methylated with methanol/BF_3_ (Sigma-Aldrich, Milan, Italy) at 90 °C for 2 h. Fatty acid methyl esters were extracted 3-fold with hexane, concentrated under nitrogen stream and analyzed using capillary gas chromatography as previously described [45]. The amount of each considered fatty acid was calculated as nmol/10^6^ cells. The degree of unsaturation (unsaturation index, U.I.) was calculated as the sum of each unsaturated fatty acid concentration, multiplied by its double bond number and divided by the total unsaturated fatty acid concentration.

### 3.3. Membrane Fluidity and Peroxidation Potential

The effects of the enrichment of HMEC with n-3 LC-PUFA on both peroxidation potential and membrane properties were determined after 24 hours of incubation performed, as described above. Membrane fluidity status was determined measuring the anisotropy of the fluorescent probe 1,6-diphenyl-1,3,5-hexatriene (DPH) [32,46]. The DPH probe was excited at a wavelength of 340 nm, and the emission wavelength was set at 420 nm. Samples were then excited with vertically polarized light and the intensity of the emitted light, vertically (I_v_) and horizontally (I_h_) polarized, were measured. Anisotropy (rs) was calculated with the equation: r_s_ = I_v_ − I_h_/I_v_ + 2I_h_

Membrane fluidity is a biophysical property of membranes that quantitatively expresses the mobility and the rate of membrane lipid molecule rotational motion. The anisotropy of DPH is inversely related to membrane fluidity when inserted between the outermost portions of the fatty acyl chains.

The sensitivity to peroxidation was determined by detecting the kinetics of intracellular reactive oxygen species formation after adding 2,2′-azobis(2-amidinopropane) dihydrochloride (AAPH) at 37 °C, using dichlorofluorescin diacetate as probe [47]. AAPH is a thermolabile compound that can give rise to a flux of hydro-soluble free radicals. The slope of the kinetics of the peroxidation curves was used as an index of peroxidation sensitivity.

### 3.4. Na,K-ATPase Activity

The effects of n-3 LC-PUFA enrichment on the activity of the membrane enzyme Na,K-ATPase were measured by determining its inhibition by oubain (1 mM), as previously described [48]. This concentration guarantees the inhibition of both the isoforms of human endothelial cell sodium pump [16]. Briefly, after 24 h of incubation performed as described above, the cells were trypsinized, centrifuged (Megafuge 8 series, Heraeus™, Thermo Scientific, Monza, Italy) at 120× *g* for 5 min and washed twice with PBS. To lyze the cells, HMEC were resuspended in 25 volumes of cold hypotonic buffer (10 mM Tris-HCl, pH 7.4) and incubated on ice for 5 min. Lysates were then centrifuged for 30 min at 100,000× *g*, 4 °C (Optima Max, Beckman Coulter, Cassina De' Pecchi, Milan, Italy). The membrane pellet was then re-suspended in cold hypotonic buffer and the centrifugation step was repeated 3 times. Protein concentrations were determined by the method of Lowry et al. [49] using bovine serum albumin (Sigma-Aldrich, Milan, Italy) as a standard. ATP-ase activity was measured by preincubating the cells at 37 °C for 10 with 92 mM tris-HCl pH 7.4 containing 100 mM NaCl, 20 mM KCl, 5 mM MgSO_4_,1 mM EDTA (Sigma-Aldrich, Milan, Italy), with or without 1 mM oubain (Sigma-Aldrich, Milan, Italy). After incubation at 37 °C for 10 min with 4 mM vanadate-free ATP (Sigma-Aldrich, Milan, Italy), the reaction was stopped by adding ice-cold trichloroacetic acid (final concentration 5%). Cells were then centrifuged for 10 min at 5,500× *g* at 4 °C and the supernatant was used for the determination of inorganic phosphate (Pi) [50]. Pump activity was calculated as the difference between the Pi concentrations, obtained in the presence or in the absence of oubain, and expressed as nmol Pi/hour/mg protein.

### 3.5. Statistical Analysis

All the measurements were repeated at least three times in triplicate. Data are expressed as the mean ± SD. The linear relationships between n-3 LC-PUFA concentration and EPA and DHA incorporation, membrane fluidity and peroxidation potential were assessed using Pearson’s correlations. Linear regression curves were compared by one-way ANOVA. The significance of the difference between the means was assessed by unpaired *t*-test. A *p*-value ≤ 0.05 was considered statistically significant. Statistical analysis was performed by using StatistiXL software (version 1.5; StatistiXL, Nedlands, Australia).

## 4. Conclusions

Increased dietary intake of long-chain n-3 polyunsaturated fatty acids has been shown to be beneficial for the vascular tree [51]. However, controversies exist on this topic [10]. Recently, EPA was shown to improve endothelial function [52]. This is crucial since endothelial cells are considered the gatekeeper of vascular health. Whereas high concentrations alter membrane properties and inhibit Na,K-ATPase pump activity, our study highlights that a low concentration, namely 3.75 µM/mL, of EPA and DHA minimizes peroxidation potential and optimizes activity. While this concentration will be useful for designing new *in vitro* and *in vivo* studies that might reconcile the contrasting reports available in the literature, it should be noted that our results are complex to translate into clinical observation because the normal range of EPA and DHA fall within a very wide range.
